# Thermal ocular injury associated with retinal necrosis and vitreous inflammation: a case report

**DOI:** 10.3389/fmed.2025.1511514

**Published:** 2025-06-25

**Authors:** Ruiping Gu, Yue Guo, Boya Lei, Rui Jiang

**Affiliations:** ^1^Department of Ophthalmology, Eye and ENT Hospital of Fudan University, Shanghai, China; ^2^Ocular Trauma Center, Eye and ENT Hospital of Fudan University, Shanghai, China; ^3^Shanghai Key Laboratory of Visual Impairment and Restoration, Fudan University, Shanghai, China; ^4^NHC Key Laboratory of Myopia (Fudan University), Laboratory of Myopia, Chinese Academy of Medical Sciences, Shanghai, China; ^5^Department of Ophthalmology, Peking University People’s Hospital, Beijing, China

**Keywords:** thermal retinal burns, retinal necrosis, endophthalmitis, sclera ischemia, vitrectomy

## Abstract

**Background:**

A case of thermal ocular injury associated with retinal necrosis and vitreous inflammation is reported.

**Case presentation:**

A 53-year-old man’s right eye was hit by a heated screw, which embedded directly into the nasal orbit. Two weeks after the foreign body was removed, serious vitreous inflammation and a large yellow–white lesion on the nasal peripheral retina, which resembled a subretinal abscess, were observed. Endophthalmitis could not be ruled out and vitrectomy was performed. During vitrectomy, we identified the yellow–white lesion as retinal necrosis and edema, with localized retinal detachment within the lesion. After surgery, there was significant macular atrophy with poor vision.

**Conclusion:**

Thermal retinal burns must be differentiated from infectious endophthalmitis to ensure timely anti-inflammatory treatment.

## 1 Introduction

Ocular burns represent about 10% of ocular trauma (1, 2) and are classified according to their etiology as either chemical (acid or alkali) or thermal injuries (3). Thermal injuries are caused by radiant energy (heat, electricity) or ultraviolet (UV) radiation (3). They are potentially blinding and constitute true emergencies. Thermal injuries are less common than chemical injuries and cause a different type of ocular insult, pose different post-injury problems, and require different treatment approaches. Work-related accidents are the main sources of ocular burns. The severity of the injury is determined by the depth and degree of the epithelial damage and the limbal ischemia (4). The majority of ocular thermal injuries caused by radiant energy involve the anterior segment of the eye and the accessory apparatus, such as the anterior sclera, conjunctiva, cornea, and eyelids (4–6). There likely have not been reported cases of thermal injury to the retina for several reasons. First, ocular radiant damage involving the posterior segment is rare. Second, ocular burns are often accompanied by severe corneal opacity, which blocks any observation of the fundus. Here we report a case of a thermal orbital injury caused by a hot screw, which was accompanied by a serious vitreous inflammatory reaction and localized retinal necrosis.

### 1.1 Case report

**FIGURE 1:**
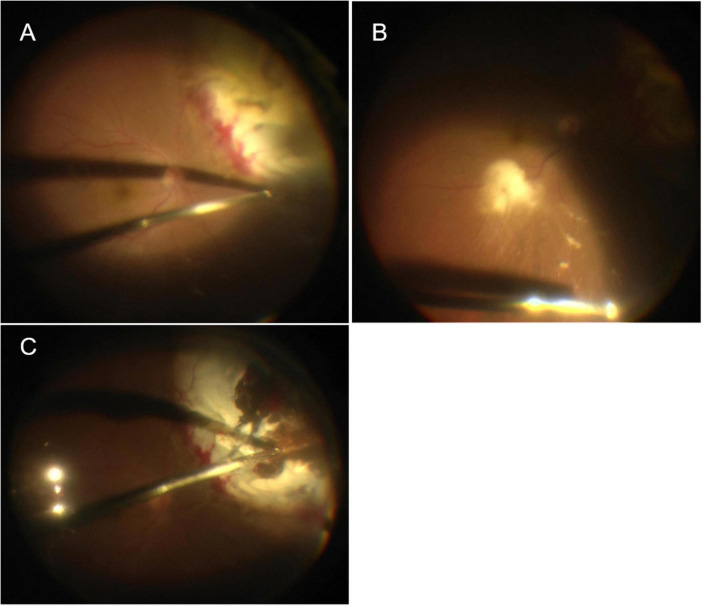
**(A)** The orbital CT showed a foreign body, about 20 × 12 mm in size, partially involving the nasal ocular wall; **(B,C)** the foreign body was removed directly from the orbit outside ocular and the ocular wall was intact after removal.

**FIGURE 2:**
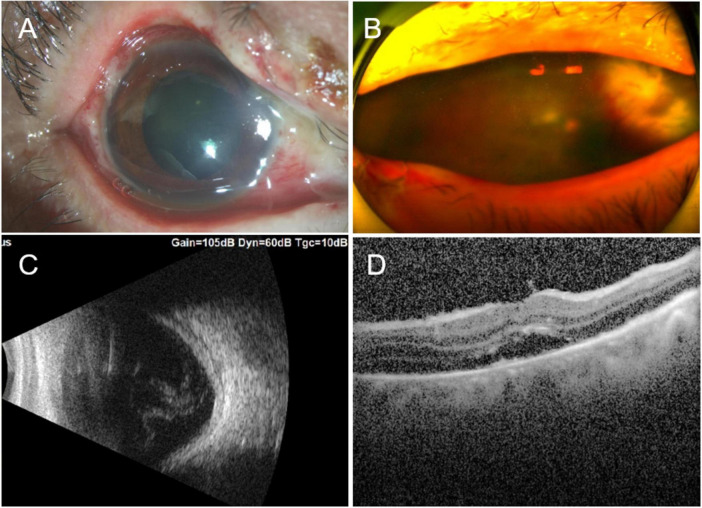
**(A)** Two weeks after the ocular burn, edematous eyelids, nasal limbal ischemia (Roper-Hall category II), mixed congestion of the conjunctiva, and corneal edema were observed, together with panel **(B)** vitreous opacity (3+) and a large yellow–white lesion on the nasal peripheral retina, which resembled a subretinal abscess. **(C)** B-scan ultrasound showed ocular edema and vitreous opacity. **(D)** OCT showed subretinal fluid in the macula. OCT, optical coherence tomography.

**FIGURE 3:**
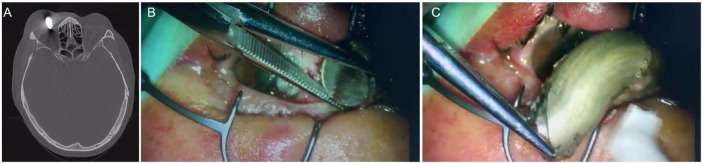
**(A)** Yellow–white lesion on the superior nasal peripheral retina, which looked like a subretinal abscess but was actually retinal necrosis and edema, with localized retinal detachment. **(B)** Flaky yellowish-white material seen next to the infratemporal vascular arch and the inferior area was suspended necrotizing tissue. **(C)** The necrotic detached retina was removed.

**FIGURE 4:**
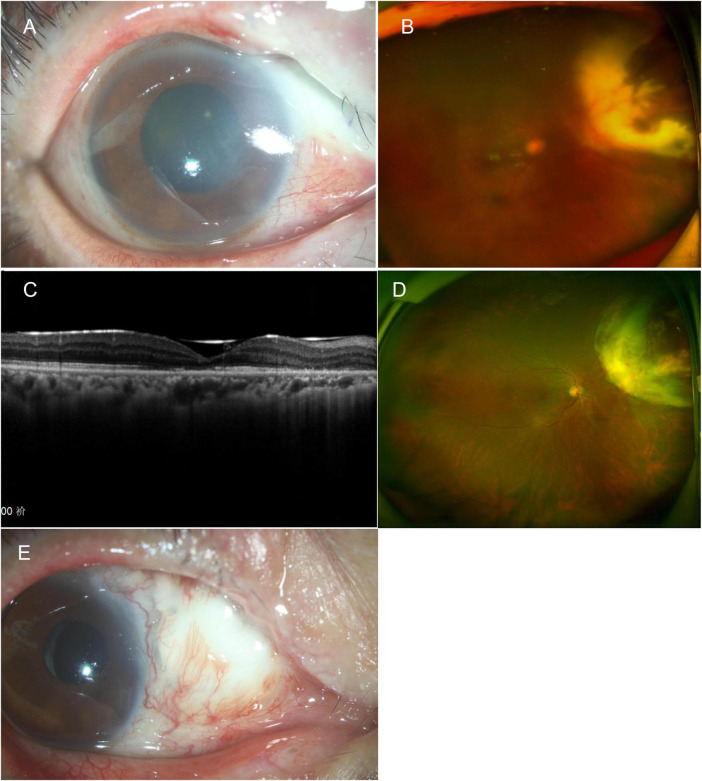
**(A)** One week after vitrectomy, anterior segment photography shows pallor and ischemia of the superior nasal sclera. **(B)** Ultra-widefield fundus photography shows intraocular silicone oil tamponade with yellow-white lesion in the superior nasal area. **(C)** 6 months post vitrectomy, the OCT shows atrophy of the outer layer of the macula. **(D)** The peripheral nasal retina showed atrophy, and the atrophy lesion remained stable following removal of the silicone oil. **(E)** 1 year post vitrectomy, the sclera in the superior nasal area became thin and developed a perforation. An allogeneic scleral transplant was eventually performed. OCT, optical coherence tomography.

A 53-year-old man came to the emergency department with sudden-onset eye pain, visual loss, and an inability to open the right eye for 6 hours. About 6 h earlier, a hot screw on the production line accidentally broke and popped off and hit the patient’s right eye. His vision was hand motions. A slit lamp examination detected grossly swollen eyelids, a cylindrical metallic foreign body with a diameter of about 10 mm outside the nasal conjunctiva, intact epithelium, nasal corneal stromal edema, nasal limbal ischemia (Roper-Hall category II), (7) and a quiet anterior chamber that was negative for keratic precipitates but positive for cell infiltration (1 + cell), anterior chamber flare (1 + flare) and mild cataract. Examination of the posterior segment was unsuccessful. Intraocular pressures (IOP) measured 14 mmHg on the right. Orbit computed tomography showed a foreign body, about 20 mm × 12 mm in size, involving the nasal orbit (Figure 1A). The foreign body was surgically removed directly from the nasal orbit. The eye wall was intact and no sutures were needed (Figures 1B, C and Supplementary Video 1). The foreign body was sent for bacterial culture and drug sensitivity testing. After surgery, ceftazidime (2 g) was given intravenously for 3 days, and topical treatment with prednisolone acetate, atropine, and levofloxacin eye drops was administered (four times a day). The intraocular pressure (IOP) on right eye was 31 mmHg 2 days later and returned to 16 mmHg on the third day by using antiglaucoma medications (brimonidine tartrate and brinzolamide eye drops). Two weeks after surgery, the edema of the upper and lower eyelids of the right eye improved significantly. A pale conjunctiva and sclera on the nasal side, mixed congestion of the remaining conjunctiva, corneal edema, anterior chamber cell (1+), anterior chamber flare (1+), mild cataract, vitreous opacity (3+), and a large yellow–white lesion on the nasal peripheral retina that resembled a subretinal abscess could been seen (Figures 2A, B). The B-scan ultrasound showed ocular edema and vitreous opacity (Figure 2C), and optical coherence tomography (OCT) showed subretinal fluid in the macula (Figure 2D). Bacterial culture of the foreign body resulted in the growth of *Staphylococcus epidermidis*. Because endophthalmitis could not be ruled out, vitrectomy was performed under general anesthesia. During vitrectomy, we found that the yellow–white lesion that looked like a subretinal abscess on the nasal peripheral retina was actually retinal necrosis and edema, with localized retinal detachment in the lesion (Figure 3A). A flaky yellowish-white material was observed next to the infratemporal vascular arch and the inferior area suspended necrotizing tissue (Figure 3B). A core vitrectomy and peripheral shaving were performed, together with the resection of the necrotic detached retina (Figure 3C), with silicone oil tamponade after gas–liquid exchange. Postoperatively, the lesion was stabilized and no retinal detachment occurred. Bacterial and fungal smears and cultures of vitreous samples were negative. One-week post-vitrectomy, the patient showed significant improvement in conjunctival congestion, while exhibiting pallor and ischemia in the superior nasal sclera (Figure 4A). Additionally, ultra-widefield fundus photography revealed intraocular silicone oil tamponade with a yellow-white lesion in the superior nasal area (Figure 4B). By the 6-month follow-up, atrophy was observed in the superior nasal peripheral retina, and the outer layers of the macula appeared atrophic on OCT (Figure 4C). Removal of the silicone oil and was successful, and the peripheral nasal atrophy lesion remained stable post-removal (Figure 4D). Due to the worsening of the cataract, phacoemulsification combined with intraocular lens implantation was also performed during the silicone removal. The patient’s best corrected visual acuity (BCVA) was 0.05, and his IOP remained within normal range following the surgery. One year post vitrectomy, the sclera in the superior nasal area became thin and developed a perforation. An allogeneic scleral transplant was performed (Figure 4E). The patient’s left eye exhibited no abnormalities throughout the treatment process, with intraocular pressure maintained within the normal range and a best-corrected visual acuity (BCVA) of 1.0.

## 2 Discussion

Thermal ocular burns are one of the most urgent ophthalmic emergencies, may result in permanent damage, and in some cases, blindness. Therefore, they require active management. Because the lid reflex is extremely rapid, the eyelid provides protection for the eye itself. What’s more, posterior segment sequelae are often difficult to assess in thermal ocular injuries, because corneal opacification frequently precludes visualization of the retina. In the present case, a thermal retinal injury induced a serous vitreous inflammatory response and retinal necrosis.

Thermal injuries to the eye generally occur as a result of exposure to scalding liquid, direct flame, or items such as cigarettes or curling irons (5). In our case, a hot screw flew out and hit the patient’s right eye during a work-related accident. The rapid movement of the screw caused it to embed directly into the nasal orbit, burning the surrounding tissues, including the eyelids, conjunctiva, sclera, and retina. Because the screw was embedded directly in the deep tissue, the corneal involvement was mild and the clear cornea allowed us to observe the changes in the fundus. There has been a similar reported case of an alkali burn in which the alkaline substance penetrated through the conjunctiva, sclera, choroid, and retina, resulting in full-thickness necrosis (8). Much like chemical burns, the severity of thermal eye burns is related to the duration of exposure and the nature of the causative agent. In our case, the hot screw led to the necrosis of the adjacent conjunctiva, sclera, choroid and retina. Toxic substances, such as prostaglandins, superoxide radicals—and presumably histamine, angiotensin, leukotrienes, and other molecules—are released from the necrotic cells (9, 10). When the cytokines diffuse into the surviving tissues, an inflammatory response is initiated. In severe burns, a severe and long-term inflammatory process is initiated. Two weeks after this burn injury, the reaction in the anterior chamber was relatively mild, but serious vitreous opacity (3+) and a large yellow–white lesion on the nasal peripheral retina, which looked like a subretinal abscess, were observed. Bacterial culture of the foreign body showed growth of *S. epidermidis*. Because endophthalmitis could not be excluded, vitrectomy was performed. During vitrectomy, we found that the yellow–white lesion was retinal necrosis rather than a subretinal abscess. The suspended necrotic retinal fragments released to the inferior retinal surface looked very similar to the bacteriophages commonly seen in patients with endophthalmitis (11). The ocular inflammation improved rapidly after vitrectomy, which may be attributable to the complete removal of the various proinflammatory factors released into the vitreous cavity by the necrotic tissue. Also, the silicone oil tamponade was likely mainly responsible for the rapid reduction of the ocular inflammation post vitrectomy.

For all thermal injuries of the eye, the first priority is to remove the individual from the source and to cool the tissues as rapidly as possible. For emergency treatment, the artificial tears, topical prednisolone, analgesics and antibiotic eye drops or systemic antibiotics were recommended. Further therapeutical procedures are applied according to the extent of the damage. The patient’s condition was finally stabilized and the silicone oil was successfully removed 6 months later. However, a review of the patient’s treatment raises one question. Is vitrectomy the best choice in this situation, and can corticosteroids be used as an alternative? We selected vitrectomy because the patient’s primary surgery was performed by another surgeon, the intraoperative situation was not well understood at that time, and the eye’s clinical manifestations were highly suggestive of endophthalmitis. Despite there was no thermal necrosis to the macula, on the OCT in Figure 2D, the macula was involved with a serous retinal detachment, presumably from the inflammatory cytokines released by the necrotic retina. Further study is warranted in animal models of thermal retinal necrosis to find the optimal treatment regimen, steroids or vitrectomy, to see which methodology has the best treatment outcome.

## 3 Conclusion

This is the first reported case of a thermal retinal burn. It must be differentiated from infectious endophthalmitis to ensure timely anti-inflammatory treatment, which is critical.

## Data Availability

The original contributions presented in this study are included in this article/Supplementary material, further inquiries can be directed to the corresponding author.

## Supplementary material

Video 1 The surgery to remove the foreign body under local anesthesia.
